# Electronic Characterization of a Charge-Transfer Complex Monolayer on Graphene

**DOI:** 10.1021/acsnano.1c01430

**Published:** 2021-05-24

**Authors:** Avijit Kumar, Kaustuv Banerjee, Mikko M. Ervasti, Shawulienu Kezilebieke, Marc Dvorak, Patrick Rinke, Ari Harju, Peter Liljeroth

**Affiliations:** † School of Basic Sciences, Indian Institute of Technology Bhubaneswar, Jatni, 752050 Khurda, India; ‡ Department of Applied Physics, Aalto University, FI-00076 Aalto, Finland; ¶ Varian Medical Systems Finland, FI-00270 Helsinki, Finland

**Keywords:** scanning tunneling microscopy (STM), charge-transfer complex, F_4_TCNQ, TTF, epitaxial graphene, charge density wave (CDW)

## Abstract

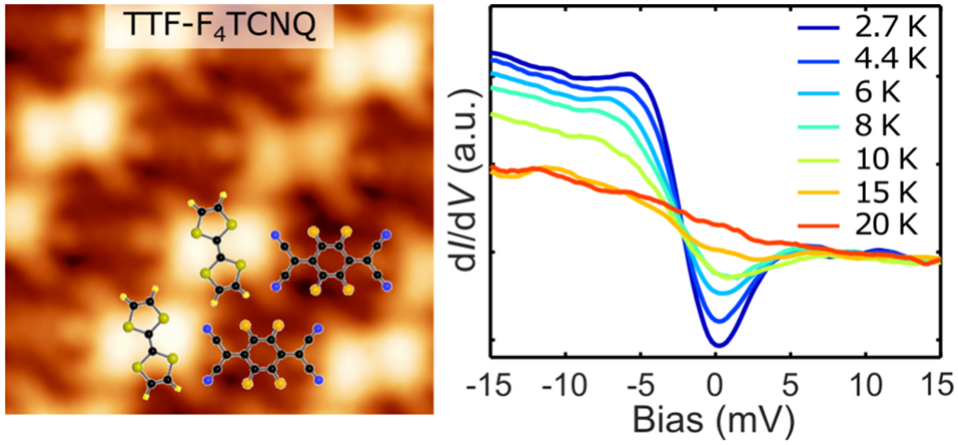

Organic charge-transfer complexes (CTCs) formed by strong electron acceptor and strong electron donor molecules are known to exhibit exotic effects such as superconductivity and charge density waves. We present a low-temperature scanning tunneling microscopy and spectroscopy (LT-STM/STS) study of a two-dimensional (2D) monolayer CTC of tetrathiafulvalene (TTF) and fluorinated tetracyanoquinodimethane (F_4_TCNQ), self-assembled on the surface of oxygen-intercalated epitaxial graphene on Ir(111) (G/O/Ir(111)). We confirm the formation of the charge-transfer complex by d*I*/d*V* spectroscopy and direct imaging of the singly occupied molecular orbitals. High-resolution spectroscopy reveals a gap at zero bias, suggesting the formation of a correlated ground state at low temperatures. These results point to the possibility to realize and study correlated ground states in charge-transfer complex monolayers on weakly interacting surfaces.

Organic charge-transfer complexes (CTCs) formed by electron-donor and -acceptor molecules are an intriguing and broad class of materials that can exhibit phenomena related to strong electron correlations and electron–phonon coupling such as charge and spin density waves, Mott metal–insulator transitions, charge ordering, spin-liquid phases, and superconductivity.^1−6^ In bulk CTC crystals, donor and acceptor molecules typically stack in rows that maximize π–π electronic overlap along the rows only.^7^ This anisotropy in the overlap results in pseudo-one-dimensional electronic dispersion, providing a suitable platform to investigate low-dimensional, as well as low-energy, physics. Despite the broad spectrum of intriguing physical phenomena that have been reported in bulk CTCs, their two-dimensional (2D) films have been much less studied.^8−16^ In particular, the studies have been confined to metal substrates, which strongly interact with the molecular layer and mask the intrinsic electronic properties of the CTCs.

The CTC formed out of tetrathiafulvalene (TTF) and tetracyanoquinodimethane (TCNQ) molecules is an archetypal example of a CTC. It possesses the highest bulk conductivity reported so far in a CTC and has been studied in detail.^1,7,16−18^ Another widely studied system is formed by the Bechgaard salts consisting of small, planar organic molecules acting as an electron donor combined with an electron-accepting small inorganic molecule. These materials are one of the most prominent examples of organic superconductors.^1,11^

The properties of 2D films of these CTCs on metallic substrates can be strongly influenced by the underlying substrate. For example, it is possible to form films with other than 1:1 stoichiometry.^12−14^ In some cases, the effect of the substrate can be limited to doping of the film, *e*.*g*., in the case of the organic superconductor BETS_2_GaCl_4_ monolayer on Ag(111).^11,15^ On the other hand, the substrate interaction can completely dominate the low-energy electronic properties. On Au(111), TTF-TCNQ molecular states of the CTC hybridize with the metal states to form dispersive interface states.^8^ Further, the unpaired electron of TCNQ molecules on the Au(111) surface exhibits the many-body Kondo effect due to screening by the substrate conduction electrons.^9^ Thus, the electronic properties of a CTC, especially close to the Fermi energy, can be strongly perturbed by the metal substrate, prohibiting the study of intrinsic electronic properties of the CTC. Therefore, preparing 2D films of CTCs on weakly interacting substrates is extremely desirable. Epitaxial graphene grown on Ir(111) has been shown to decouple the adsorbate layer from the underlying metal substrate, allowing investigation of intrinsic electronic properties of the adsorbate layers.^19,20^

Here, we a present a low-temperature scanning tunneling microscopy (LT-STM) study of a 2D CTC of TTF and fluorinated TCNQ (F_4_TCNQ) self-assembled on the surface of oxygen-intercalated epitaxial graphene on Ir(111) (G/O/Ir(111)). Sequential deposition of the molecules on this surface leads to the formation of rotationally identical domains of CTCs with alternating rows of TTF and F_4_TCNQ lying parallel to the surface. The frontier molecular orbitals of the molecular species in the CTC, as found from scanning tunneling spectroscopy (STS), indicate charge transfer between TTF and F_4_TCNQ molecules. High-resolution tunneling spectra exhibit a dip at Fermi energy below a temperature of 20 K that may be attributed to the formation of a correlated ground state in the CTC monolayer.

## Results and Discussion

**Figure 1:**
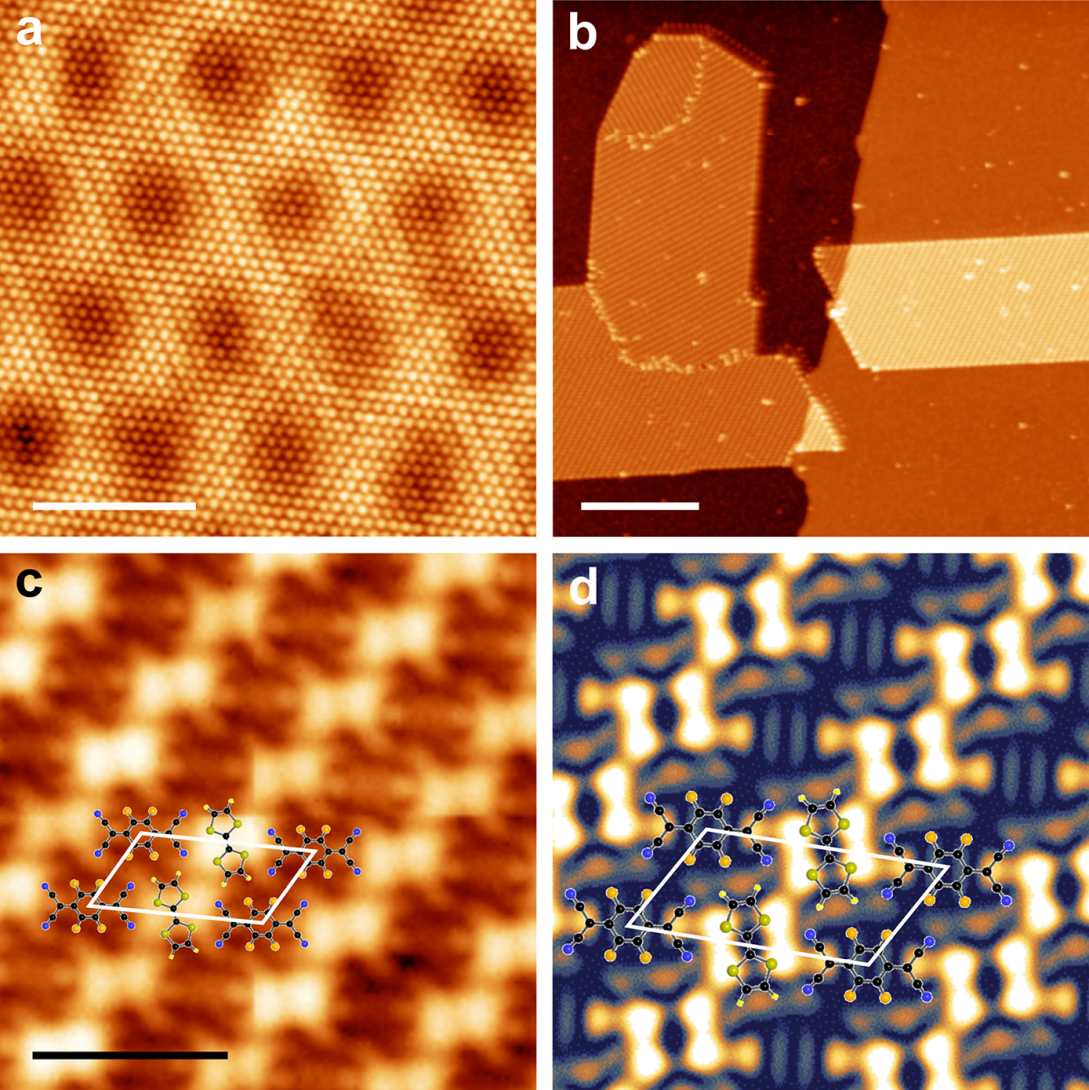
Assembly and structure of the CTC on oxygen-intercalated graphene. (a) STM topography image of oxygen-intercalated graphene on Ir(111). The additional superstructure apart from the moiré is due to reconstruction of subsurface oxygen. The scale bar is 3 nm. Imaging parameters: 1.2 nA and 10 mV. (b) Few large islands of CTC on the G/O/Ir(111) surface showing various domains and the domain boundaries. The scale bar is 30 nm. Imaging parameters: 0.4 pA and 0.75 V. (c) Zoomed-in STM image of the CTC showing the arrangement of TTF and F_4_TCNQ molecules. Each molecule forms a row next to the row of the other molecule. A molecular structure along with a unit cell is overlaid to elucidate the molecular arrangement within the unit cell. The scale bar is 2 nm. Imaging parameters: ∼5 pA and 0.1 V. (d) DFT-simulated STM image of the CTC close to the Fermi energy resembling the recorded topography closely. Molecular structure and unit cells are overlaid for clarity.

Figure 1 describes the assembly and structure of the TTF-F_4_TCNQ CTC on a G/O/Ir(111) surface. The sample preparation is described in detail in the Methods section. Briefly, we grow a near-monolayer coverage of graphene on Ir(111) by a combination of temperature-programmed growth (TPG) and chemical vapor deposition (CVD), as described previously,^21−23^ followed by oxygen intercalation to electronically decouple graphene from the underlying substrate.^24^ Finally, the molecules are deposited at low temperatures (≈ 100 K), followed by annealing at room temperature for 15–45 min to allow the formation of highly ordered CTC islands.

Figure 1a shows an STM topography image of oxygen-intercalated graphene on Ir(111). The surface contains the periodic moiré pattern of a G/Ir(111) surface with a periodicity of 25.4 Å. The additional superstructure visible on the surface is due to patches of (2 × 1) reconstruction of subsurface oxygen, which is consistent with an earlier report.^24^ Oxygen intercalation leads to decoupling of graphene from Ir, which is indicated by the short-range d*I*/d*V* spectroscopy of the surface showing a phonon gap of ∼160 mV^25,26^ (see Supporting Information (SI) Figure S1a). Oxygen intercalation also results in strong p-doping of graphene by ∼0.5 eV,^27^ which increases the work function to ∼5.1 eV. This can be independently verified by measuring d*I*/d*V* spectra at high bias with the feedback loop on; here, the field-emission resonances allow estimating the substrate work function^28−31^ (see SI Figure S1b).

Figure 1b shows an STM topograph of large islands of ordered CTCs assembled on a G/O/Ir(111) surface. The long-range ordering is the result of the postdeposition room-temperature annealing; directly after the low-temperature deposition, we observe disordered islands on the surface (see SI Figure S2). The CTC islands grow across the step edges in a carpet-like fashion^32,33^ and contain various domains rotated with respect to each other. Analysis of several images reveals a total of six domain orientations rotated with respect to each other in multiples of 30°. Figure 1c shows a zoomed-in STM image to identify arrangement of TTF and F_4_TCNQ molecules within the CTC islands. As evident from the STM image, there are two different rows of molecules: one is composed of TTF and the other of F_4_TCNQ molecules. Rows of TTF and F_4_TCNQ are lying alternately on the surface. The molecular structure obtained from density functional theory (DFT) calculations (see below) has been overlaid on the STM image for clarity. The molecular rows are found to be at an angle of ±12° compared with graphene’s zigzag direction for each domain. The unit cell of the CTC is shown by a parallelogram with lattice parameters *a* = 18.5 (±0.5) Å, *b* = 9.5 (±0.5) Å, and θ = 56 (±2)°. This is the most common phase we observe for this stoichiometry ((F_4_TCNQ)_1_(TTF)_1_) of the molecules. At a slightly different stoichiometry ((F_4_TCNQ)_*x*_(TTF)_*y*_), we have observed a checkerboard phase of the CTC where only F_4_TCNQ rows are present and TTF molecules are dispersed across in a checkerboard fashion (see SI Figure S3).

In order to further elucidate the structure of the molecular layer, we carried out a broad structural search for different possible geometries using DFT (see Methods for details). We performed full structural relaxations of 300 CTC monolayers sampled by varying intermolecular distances, bond angles, and alignment with respect to the underlying graphene. The initial structures are systematically generated but done “by hand” without any input from machine learning or structure search algorithms.^34,35^ After relaxation, the structures are sorted by formation energy. One of the low-energy conformations closely matches the experimental structure in terms of both the unit cell dimensions (*a* = 17.78 Å, *b* = 8.89 Å, θ = 60°) and the relative orientation with respect to the graphene lattice (13.89°). A DFT-simulated STM image (at the Fermi energy) is shown in Figure 1d for the optimized geometry; it closely resembles the STM image shown in Figure 1c.

We have also looked at the assembly of single-component F_4_TCNQ and TTF layers on the G/O/Ir(111) surface. A sub-monolayer coverage of F_4_TCNQ molecules forms chain-like structures (in contrast to nonplanar adsorption on the G/Ir(111) surface^36^). On the other hand, TTF molecules tend to assemble in a close-packed geometry on the G/O/Ir(111) surface. The assembly of F_4_TCNQ and TTF molecules is shown in SI Figures S4 and S5.

**Figure 2:**
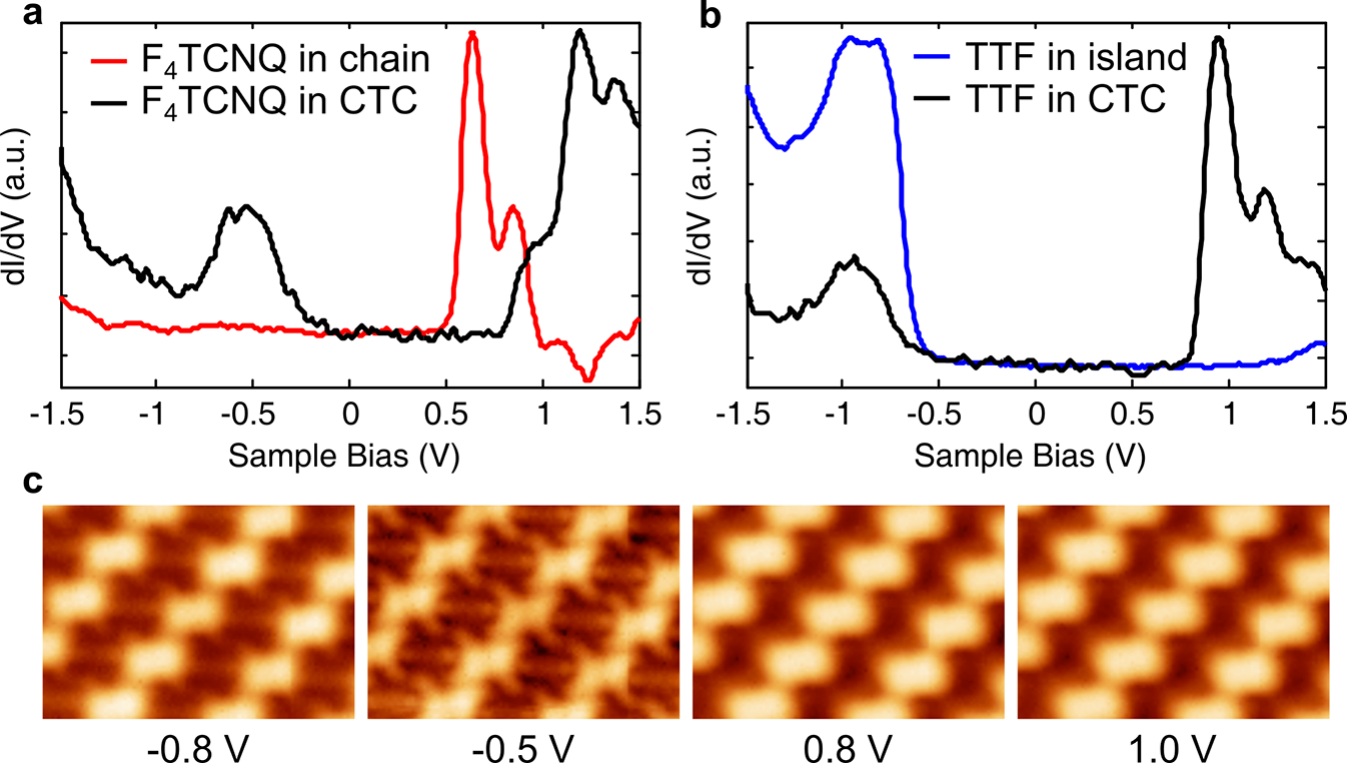
Charge transfer across the molecules. (a) Long-range d*I*/d*V* spectra on F_4_TCNQ molecules in a single-component chain on the G/O/Ir(111) surface (red line) and on the F_4_TCNQ sites in the CTC (black line). (b) Long-range d*I*/d*V* spectra on TTF molecules in a single-component assembly on G/O/Ir(111) (blue line) and on the TTF sites in the CTC (black line). (c) Bias-dependent STM images of the CTC at the sample biases indicated in the figure. The size of each image is 4.7 × 3.2 nm^2^.

Figure 2 shows the experimental verification of charge transfer between TTF and F_4_TCNQ molecules in the CTC by d*I*/d*V* spectroscopy and STM imaging. Figure 2a compares long-range d*I*/d*V* spectra recorded on F_4_TCNQ molecules in single-component chains to those recorded in the CTC. The spectrum on the molecule in the chain shows a resonance corresponding to the lowest unoccupied molecular orbital (LUMO) at 0.64 V without any features at negative bias. This indicates that the F_4_TCNQ molecules on G/O/Ir(111) are neutral, in contrast to F_4_TCNQ molecules on a G/Ir(111) surface, where they are charged at lower sites of the moiré pattern.^36^ This difference is likely due to the increased work function of graphene due to oxygen intercalation. The spectrum recorded on a F_4_TCNQ molecule in the CTC, on the other hand, shows two peaks at −0.44 and 1.2 V. Figure 2b compares the d*I*/d*V* spectrum on TTF molecules from the pristine assembly on a G/O/Ir(111) surface to that of TTF molecules from the CTC. Here, a d*I*/d*V* spectrum on the TTF molecule shows a peak at −0.8 V, corresponding to the highest occupied molecular orbital (HOMO) of a neutral TTF molecule. Despite the high work function of the surface (∼5.0 eV), the TTF molecules stay neutral. In the CTC, the spectrum on TTF molecules shows two peaks at −0.9 and 0.95 V (similar to the two peaks on an F_4_TCNQ molecule). The assignment of these peaks is done on the basis of images recorded at sample biases at −0.5 and 0.8 V. The image at 0.8 V shows a relatively prominent TTF HOMO, while the image at −0.5 V shows a relatively prominent F_4_TCNQ LUMO^36^ (see Figure 2c). Electron transfer from donor TTF to acceptor F_4_TCNQ molecules results in splitting of the TTF HOMO (−0.8 eV peak) into singly occupied (SOMO, −0.95 V peak) and singly unoccupied molecular orbitals (SUMO, 0.95 V peak). Similarly, the F_4_TCNQ LUMO (0.64 V peak) splits into SOMO (−0.44 V peak) and SUMO (1.2 V peak) after accepting an electron. Consequently, the TTF molecule acquires a positive charge, while F_4_TCNQ molecules become negatively charged in the CTC. The charge transfer between the molecules is also supported by DFT calculations, and based on Hirshfeld charge analysis^37^ it amounts to ∼0.55*e* in this configuration. Each N atom gains ∼0.2*e*, and redistribution of the remaining charge makes up the difference. The calculated band structure of the monolayer CTC (Figure 1d) is shown in SI Figure S6. From the band structure, it is evident that there is also a charge transfer from graphene to the CTC monolayer and a finite electronic coupling in the molecules along certain directions of reciprocal space (Γ–K and Γ–Y). However, the bandwidth is relatively small (∼100 meV), indicating that the coupling is quite weak.

**Figure 3:**
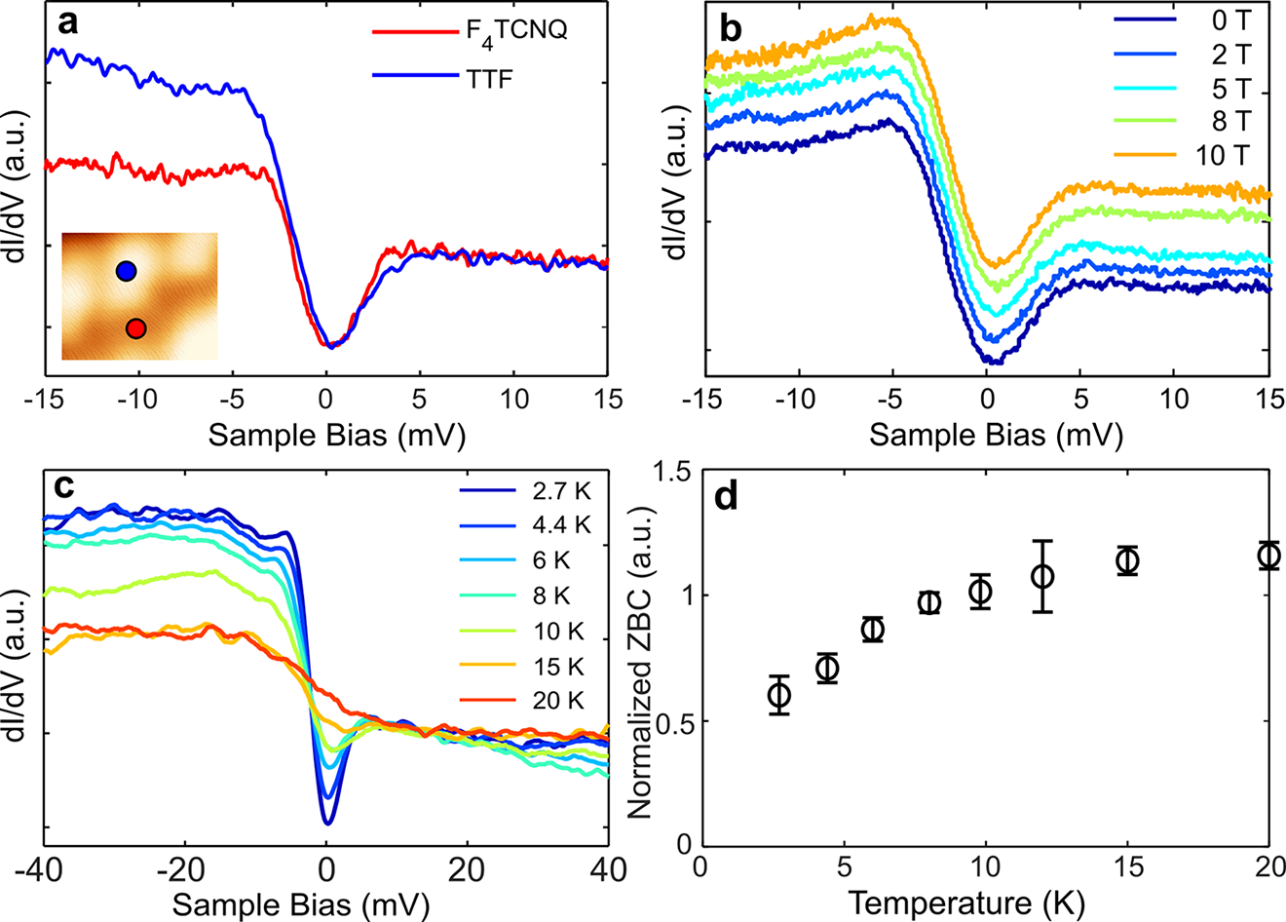
Short-range d*I*/d*V* spectroscopy on the CTC. (a) Short-range d*I*/d*V* spectra on the TTF and F_4_TCNQ sites in the CTC showing a dip at zero bias. (b) Magnetic field dependent d*I*/d*V* spectra on a TTF site in the CTC showing that the shape and size of the zero-bias dip do not change with magnetic field up to 10 T. (c) Temperature-dependent d*I*/d*V* spectra on a TTF site in the CTC showing that the dip is washed away with increasing temperature and the asymmetric background is also decreased at higher temperatures. (d) Temperature dependence of the zero-bias conductance (ZBC, normalized at the d*I*/d*V* at a bias of 20 mV) showing saturation at 15–20 K.

Interestingly, high-resolution d*I*/d*V* spectra on both molecules contain a dip close to zero bias, which has pronounced asymmetry on TTF sites, as shown in Figure 3a. To investigate its origin, we have examined its dependence on temperature and on the out-of-plane magnetic field. Care was taken to record these spectra on the same molecule and with the same microscopic tip apex. Figure 3b shows magnetic field dependent d*I*/d*V* spectra on the TTF sites of the CTC lattice in the range of 0 to 10 T. There is no measurable change in either the shape and size of the dip or the observed asymmetry up to a magnetic field of 10 T. On the contrary, a clear temperature dependence is observed from Figure 3c, which shows the temperature-dependent d*I*/d*V* spectroscopy recorded on TTF sites of the CTC from 2.7 to 20 K (data on the F_4_TCNQ site are shown in the SI Figure S7a). The asymmetric dip is most prominent at the lowest temperature of 2.7 K. The dip amplitude decreases with increasing temperature, and at 20 K only a step at zero bias remains. The temperature dependence of the zero bias conductance (ZBC) extracted from these spectra clearly exhibits saturation of the ZBC at temperatures between 15 and 20 K. This change in the ZBC indicates the presence of a low-temperature-correlated state, which we discuss in more detail below.

**Figure 4:**
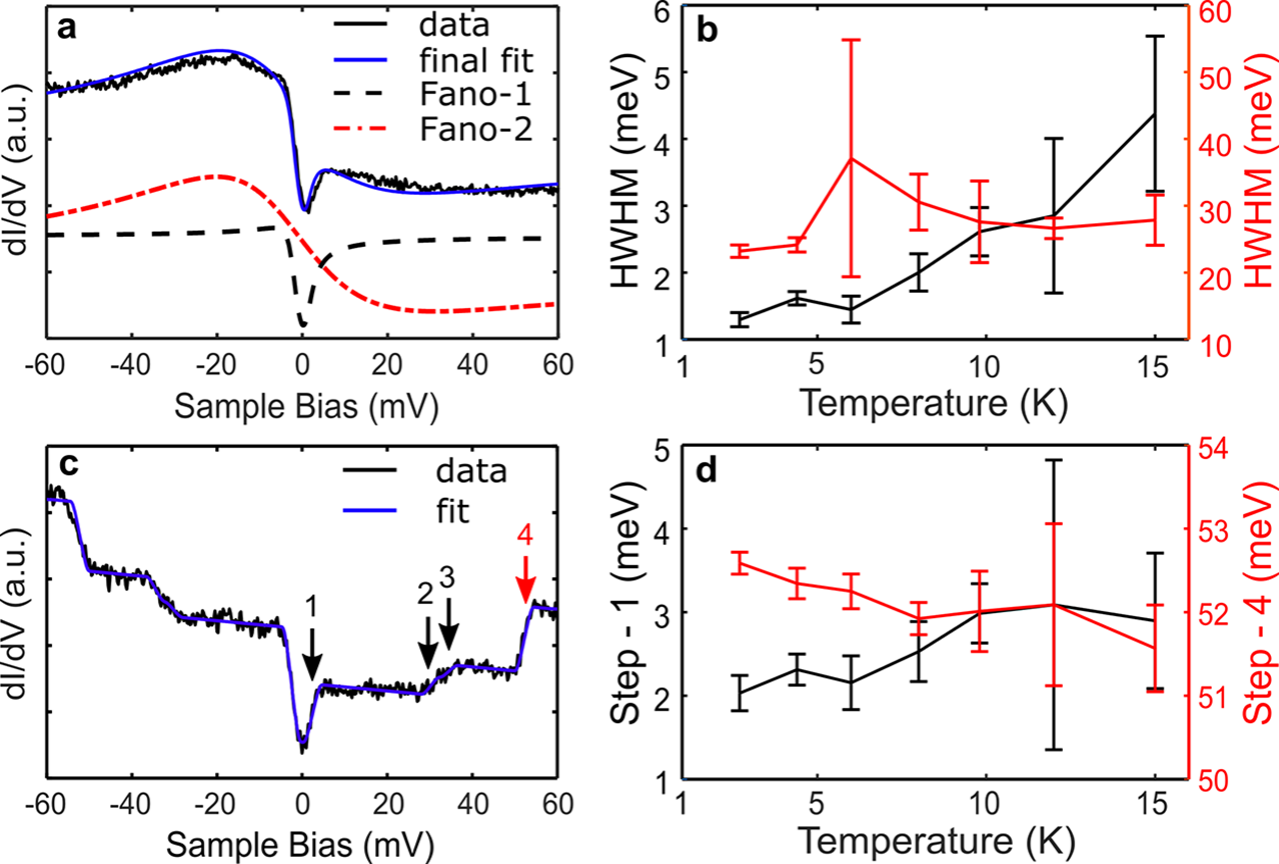
Deconvoluting the low-bias features of the d*I*/d*V* spectra. (a) Short-range d*I*/d*V* spectrum on TTF molecules. The curve has been fitted with the sum of two Fano functions: Fano-1 (broken black line) represents the central dip and Fano-2 (red line) represents the step. The final fit is indicated by a blue line. (b) Temperature-dependent evolution of HWHM extracted from the two Fano functions (Fano-1: left, Fano-2: right) from the fits. (c) Short-range d*I*/d*V* spectrum on CTC islands, recorded on an F_4_TCNQ molecule showing steps at energies ∼2 (shown by arrow 1), ∼31 (arrow 2), ∼35 (arrow 3), and ∼52 meV (arrow 4). (d) Temperature-dependent evolution of the steps at ∼2 meV (step-1: left) and at ∼52 meV (step-4: right).

The temperature-dependent spectroscopy shows that the overall asymmetry of the spectra and the amplitude of the dip decrease with increasing temperature. At 20 K, the dip feature is no longer visible while the asymmetry (a step at Fermi energy) is still present in the spectra. This suggests that the spectrum can be deconvoluted into a dip and a step; the dip vanishes at 20 K, while the step still remains visible at that temperature. The deconvolution of a spectrum measured on a TTF site in the CTC is shown in Figure 4a. The entire spectrum (note the wider bias range here compared to Figure 3a) can be well fitted (details of the fittings are described in the Methods section) by a sum of two Fano line shapes.^38^ The effect of the spectral broadening due to the bias modulation and thermal broadening has been deconvoluted (see Methods section) to obtain the intrinsic width of the line shapes. Figure 4b summarizes the temperature dependence of the half-width at half-maximum (HWHM) of the two Fano line shapes used to fit the spectra on the TTF site. The HWHM of the Fano line shape corresponding to the dip at zero bias (Fano-1) shows a clear scaling with temperature. On the other hand, the HWHM of the step-like Fano line shape (Fano-2) has a weaker temperature dependence. While the Fano line shape is taken here as a phenomenological description of the measured spectra, the choice is not completely arbitrary, as it typically arises in situations where there are two interfering tunneling pathways present. For example, it is widely observed on Kondo impurities, where the interference occurs between a direct tip–sample tunneling and tunneling path *via* the Kondo impurity.^39−42^ In fact, a spectral shape combining a step-like Fano line shape with a smaller energy gap-like feature—very similar to our measurements—has been observed on the heavy Fermion compound URu_2_Si_2_.^43^ There, the spectral response was explained by a combination of Kondo screening of the uranium f-electrons and the gap-like feature resulting from a transition to a hidden order phase at low temperatures.

Intriguingly, the d*I*/d*V* spectra recorded on the F_4_TCNQ molecules of the CTC (Figure 4c; the bias range is again wider than in Figure 3a) show additional step-like features at higher biases, *viz*., at ±31, ±35, and ±52 mV. These steps can be attributed to inelastic electron tunneling processes similar to molecular vibrations of negatively charged F_4_TCNQ molecules.^9,44^ The tunneling electrons can excite a molecular vibration once the sample bias matches the energy of the corresponding vibrational mode.^45−47^ The inelastic process corresponds to opening of an additional tunneling channel and a sudden increase in the tunneling conductance. To corroborate this picture, we assess the phonon modes for the CTC monolayer with DFT (details in the Methods). There is good agreement between the energies of the measured steps and the calculated energies of certain CTC phonon modes with a high electron–phonon coupling strength. Additionally, the calculated modes with strong coupling strength near the energies of the inelastic steps are dominated by F_4_TCNQ vibrations (see SI Figure S8 for details). This is consistent with our experiments, where we see the inelastic steps only on the F_4_TCNQ sites of the CTC.

Although DFT calculations indicate the presence of intermolecular phonon modes with energies of a few mV, the temperature dependence of the dip close to zero bias does not fit with thermally broadened inelastic steps. If we force a fit with an inelastic step to the data (feature marked with “1” in Figure 4b), the position of the fitted step would be strongly temperature dependent (Figure 4d, black symbols), which is not expected for inelastic features. The zero bias feature also washes out more quickly with temperature than what would be expected for a vibrational transition, and at 15 K or above, only an asymmetric step remains, supporting the notion that it is a result of a gap closing transition. This is illustrated in Figure S7b, which shows the expected temperature dependence for an inelastic step using the parameters extracted from the experimental spectrum acquired at *T* = 2.7 K. As can be clearly seen, the predicted trend does not match the experimental results in Figure S7a, which gives a strong indication that the zero-bias dip feature does not correspond to inelastic steps.

Considering the width of the dip, we should be able to resolve a possible magnetic field induced splitting if this feature was arising from any spin-related phenomena such as the Kondo effect or spin-flip inelastic transitions.^42,48^ However, we do not observe any such changes with a magnetic field up to 10 T, as shown in Figure 3b. While the Kondo effect has earlier been observed in a TTF-TCNQ CTC monolayer on Au(111),^9^ the Kondo coupling is expected to be generally weak on graphene.^49^ Finally, experiments on CTCs deposited on graphene directly on Ir(111) show a very similar response (see SI Figure S9). The two substrates differ significantly in terms of the doping level of graphene, which is expected to have a marked influence on the Kondo temperature.^49−51^ Further, CTCs are also known for exhibiting superconductivity. But in light of the spectroscopy measurements in high magnetic fields, a superconductivity origin of the dip at the Fermi energy is also very unlikely. One would expect either quenching or at least changes in the superconducting gap under high field. We also do not observe coherence peaks in the spectra that are usually associated with superconductivity.

**Figure 5:**
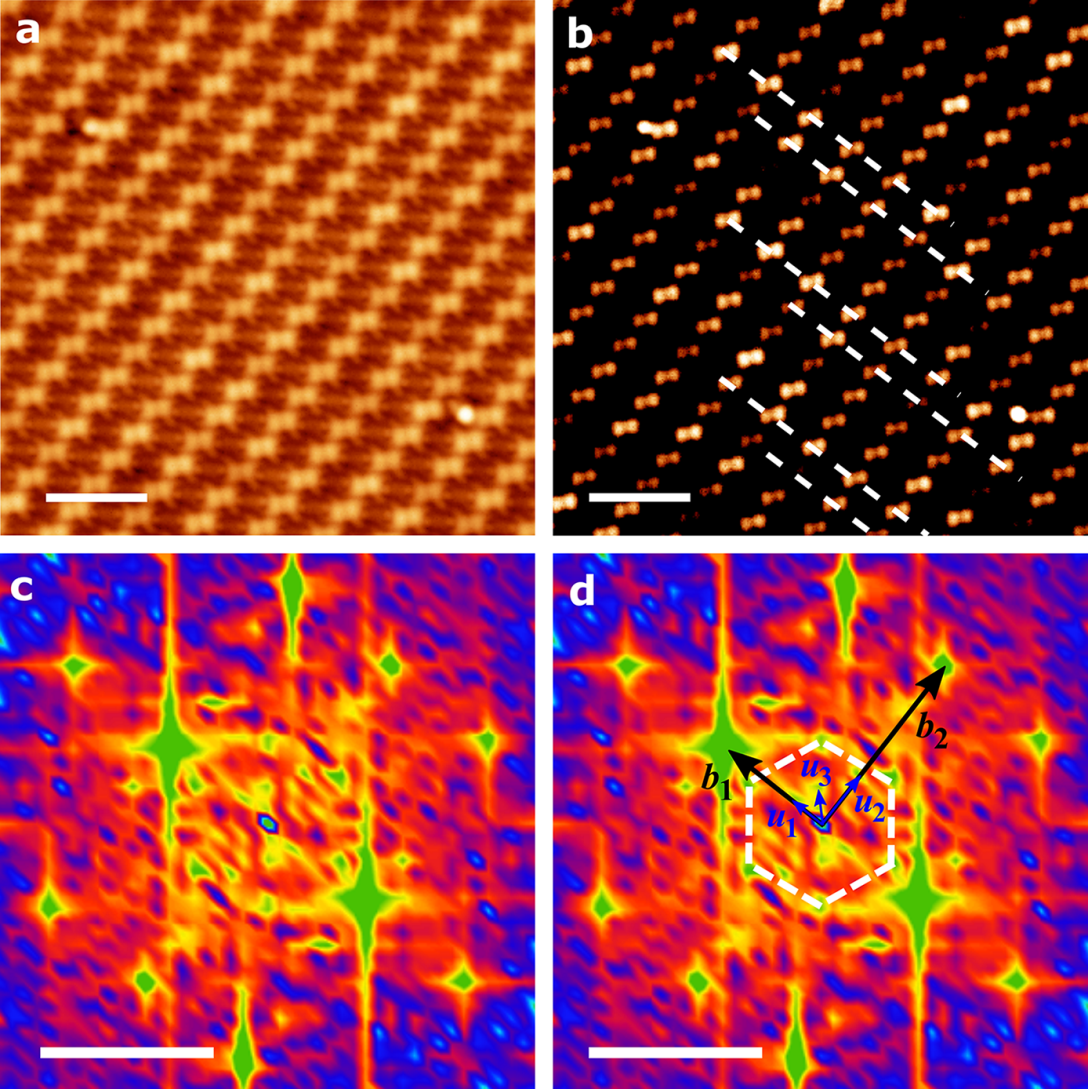
(a) STM topography image of a CTC at imaging parameters 5 pA and −500 mV. The scale bar is 3 nm. (b) Contrast-optimized version of the topography in panel (a) showing periodic topography modulations (white lines are a guide to the eyes). (c, d) Two-dimensional fast-Fourier transform (2D-FFT) of panel (a) showing features corresponding to the CTC rectangular lattice (marked by vectors ***b*_1_** and ***b*_2_**), spots due to the underlying graphene moiré (white hexagon), and charge density wave modulations by vectors ***u*_1_**, ***u*_2_**, and ***u***_3_. CDW wavelengths corresponding to ***u*_1_** and ***u*_2_** are approximately 3.25 × *l*_1_ and 3.25 × *l*_2_, while that corresponding to ***u*_3_** is ∼5 nm. The scale bar is 1 nm^–1^.

The remaining explanations consistent with the spectral feature and its dependence on magnetic field and temperature include the formation of a charge-density wave (CDW) or Peierls instability at low temperatures; these correlated ground states have been commonly observed in bulk CTC materials.^1,17^ The structure of this compound, both in bulk and in our monolayer, is anisotropic: there is much stronger electronic coupling along a certain lattice direction than in the perpendicular direction. This is also evident in the calculated band structure shown in Figure S6. This kind of anisotropic bandstructure is favorable for the formation of a CDW state, as it naturally provides Fermi surface nesting. This leads to the CDW driven by e–ph coupling, which is also in line with the picture for the bulk TCNQ-TTF phases.^1^ The temperature dependence of the ZBC (Figure 3d) clearly indicates a transition temperature of 15–20 K, which is close to the expected temperature range of a CDW or Peierls transition; for example, in the bulk TTF-TCNQ CTC this is 54 K.^52^ Finally, the ground state associated with CDW breaks the symmetry of the system and results in a superstructure arising from modulations in electron density or CTC atomic structure. Figure 5a shows an STM topography image of the CTC. A contrast-optimized version of the same image in Figure 5b shows periodic modulation of the topography, which can better be understood using 2D FFT. White lines are a guide to the eyes. Figure 5c and d show the 2D-FFT images of the topography with various spots identified. The set of spots marked by vectors ***b*_1_** and ***b*_2_** corresponds to the CTC rectangular lattice (see SI Figure S6), while the spots due to the underlying graphene moiré are indicated by a white hexagon. The set of vectors indicated by ***u*_1_**, ***u*_2_**, and ***u*_3_** indicate the presence of longer wavelength charge-density wave modulation. CDW wavelengths corresponding to ***u*_1_** and ***u*_2_** are approximately 3.25 × *l*_1_ and 3.25 × *l*_2_, while that corresponding to ***u*_3_** is ∼5 nm. Here, ***l*_1_** and ***l*_2_** are real space lattice vectors perpendicular to and along the TTF/F_4_TCNQ molecular rows, respectively. This provides further evidence of the presence of a CDW/Peierls ground state in the TTF-F_4_TCNQ CTC monolayer at low temperatures causing a gap in the density of states at the Fermi energy.

## Conclusions

In conclusion, we have synthesized a monolayer of charge-transfer complex TTF-F_4_TCNQ on a weakly interacting epitaxial graphene substrate and have investigated its intrinsic electronic properties. TTF and F_4_TCNQ molecules assemble into close-packed islands with alternating rows of TTF and F_4_TCNQ molecules in a 1:1 stoichiometry. Low-temperature STM and STS measurements confirm the formation of a charge-transfer complex with d*I*/d*V* spectra consistent with the presence of TTF cations and F_4_TCNQ anions. High-resolution spectroscopy at low temperatures and high magnetic fields show formation of a correlated ground state related to a CDW or Peirls instability with a transition temperature of 15–20 K. This work demonstrates CTC monolayers as intriguing example of two-dimensional materials with low-temperature-correlated ground states.

## Methods

### Sample Preparation

The experiments were carried out in ultra-high-vacuum (UHV), low-temperature scanning tunneling microscopes (STMs) (Createc LT-STM and Unisoku USM-1300). Both STMs are equipped with a preparation chamber and operate at a base pressure lower than 1 × 10^–10^ mbar. The sample was prepared by depositing F_4_TCNQ and TTF molecules sequentially on an oxygen-intercalated graphene on an Ir(111) substrate. The Ir(111) surface was cleaned by repeated cycles of sputtering using Ne ions at an energy of 1.5 kV and annealing at 900 °C in an oxygen environment, followed by flashing to 1300 °C. Epitaxial graphene was grown using ethylene gas with a combination of temperature-programmed growth (TPG) and CVD steps to achieve a nearly full monolayer coverage of graphene.^21−23,53^ In the TPG step, the cleaned Ir(111) substrate was exposed to the ethylene gas for 1 min at a pressure of 1 × 10^–6^ mbar followed by heating the substrate to 1300 °C. The CVD step was carried out at this temperature by exposing the substrate to ethylene gas at 3 × 10^–7^ mbar for 60 s. This gives nearly a monolayer coverage of graphene on Ir(111) (G/Ir(111)). Oxygen intercalation of G/Ir(111) (G/O/Ir(111)) was carried out by exposure of 9 × 10^4^ L oxygen at 225 °C as reported by ref (24).

The charge-transfer complex was synthesized by first depositing a ∼0.25 monolayer of F_4_TCNQ molecules on a G/O/Ir(111) surface at low substrate temperature (≈ 100 K), followed by deposition of a similar amount of TTF molecules at a similar substrate temperature. This resulted in disordered islands of CTC on the surface. The sample was annealed at room temperature for 15–45 min to allow the formation of highly ordered CTC islands. While F_4_TCNQ molecules were evaporated using a Knudsen cell heated to 92 °C, TTF molecules were evaporated from a homemade evaporator kept at a temperature of 23 °C. The deposited amounts of the two molecules were adjusted to 1:1 stoichiometry (each of them at less than a half monolayer coverage). Subsequently, the sample was transferred into the low-temperature STM housed within the same UHV system.

### STM Measurements

The STM experiments were carried out at a temperature of 4.2 K unless otherwise stated. Temperature-dependent measurements were carried out in the Createc STM, while magnetic field dependent measurements were carried out in the Unisoku STM. For the measurements at 2.7 K, the LHe cryostat of the STM was pumped, while measurements at a temperature higher than 4.2 K was achieved by heating the STM by a Zener diode installed on the STM scanner. To avoid any ambiguity, the temperature-dependent measurements were carried out on the same F_4_TCNQ and TTF molecules of the CTC assembly using the same tip. Similar precautions were taken for the magnetic field measurements as well, where the same molecules and the tip were used for the full range of the magnetic field sweep. STM measurements were carried out using mechanically cut Pt/Ir tips. d*I*/d*V* spectroscopy was performed using a standard lock-in technique, where a voltage modulation with an amplitude of 10–15 mV and 1–2 mV signal has been used for long-range and short-range spectroscopies, respectively. WSxM^54^ and Gwyddion (http://gwyddion.net/)^55^ software were used to process all the STM images.

### Fitting of the d*I*/d*V* Spectra

We use two Fano line shape functions to fit the short-range d*I*/d*V* spectrum in Figure 4a. The Fano line shape function is
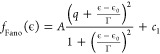
where *A* is the prefactor, ϵ is the energy, ϵ_0_ is the offset from zero, Γ is the half-width at half-maximum, *q* is the Fano parameter, and *c*_1_ is a constant background term. We first fit the step-like Fano line shape to capture the step of the spectrum (Fano-2) by excluding the central dip during the fitting. Further, we subtract the step-like Fano fit (red line in Figure 4a) from the spectrum to get a central dip, which is fitted again using a dip-like Fano line shape (Fano-1). The fitting process is repeated for all the recorded spectra at the indicated temperatures to extract HWHM for the two Fano line shapes as a function of temperature.

To fit the temperature dependence of the pair of four step features seen in Figure 4c (four on each side of zero bias), we use a series of symmetric Fermi–Dirac distribution functions as a function of energy, ϵ:
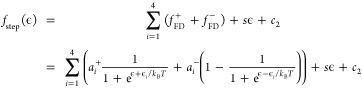
where *i* is the step number, *a*_*i*_^+^ is the amplitude of the *i*^*th*^ step for ϵ > 0, *a*_*i*_^–^ is the amplitude of the corresponding step at ϵ < 0, ϵ_*i*_ is the position of the *i*th step, *k*_B_ is the Boltzmann constant, *T* is the temperature, *s* is the slope, and *c*_2_ is a constant background term.

The recorded spectra are broadened by thermal contribution as well as the applied lock-in voltage. These effects have to be deconvoluted to get the intrinsic line shape. To correct for the lock-in modulation voltage (*V*_m_), we use the broadening function:
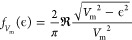
where  is the real part of a complex number. To account for thermal broadening due to the temperature (*T*) of the tip, we use the derivative of the Fermi–Dirac distribution:
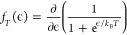
Finally, the simulated LDOS is obtained by convolving these functions, either
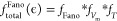
or
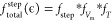
The simulated LDOS is fitted to the experimental d*I*/d*V* spectra to obtain the intrinsic line width Γ in the first case and the step positions in the second case.

### DFT Calculations

Density functional theory calculations are performed with the full potential, all-electron, numeric atom-centered orbital code FHI-AIMS.^56−59^ We use the standard FHI-AIMS “light” preconstructed basis sets of numeric atomic orbitals. Supercell calculations are performed with a 8 × 4 Γ-centered **k**-point sampling. We use the Perdew–Burke–Ernzerhof (PBE) generalized gradient approximation to the exchange–correlation functional.^60^ Van der Waals interactions are included with the pairwise Tkatchenko–Scheffler correction.^61^ Atomic forces are relaxed to less than 10^–2^ eV/Å. Vibrations are calculated with the finite difference method. Electron–phonon coupling constants are based on the electronic friction approach.^62,63^ In pursuit of open materials science,^64^ the DFT relaxed geometry of the monolayer is available in the NOvel MAterials Discovery (NOMAD) repository.^65^

## Supporting Information Available

Spectroscopy on oxygen-intercalated graphene on Ir(111); disordered islands and checkerboard phases of CTC; assembly of single-component F_4_TCNQ and TTF molecules on G/O/Ir(111); electronic band structure of CTC with or without graphene; temperature-dependent spectra, experiment and simulated; molecular vibrations of CTC; and assembly and spectroscopy of CTC grown on G/Ir(111) (PDF)
