# Supplementary material for: Electronic Characterization of a Charge-Transfer Complex Monolayer on Graphene
Source: ACS Nano. 2021 May 24;15(6):9945–54. doi: 10.1021/acsnano.1c01430 (PMC8223480; doi:10.1021/acsnano.1c01430)
Supplement: Supplementary file 1 — nn1c01430_si_001.pdf [file nn1c01430_si_001.pdf]

Supporting information for: Electronic Characterization of a Charge-Transfer Complex Monolayer on Graphene

Avijit Kumar,[*,†,‡] Kaustuv Banerjee,[‡] Mikko M. Ervasti,[‡] Shawulienu Kezilebieke,[‡] Marc Dvorak,[‡] Patrick Rinke,[‡] Ari Harju,[‡,¶] and Peter Liljeroth[*,‡]

†*School of Basic Sciences, Indian Institute of Technology Bhubaneswar, Jatni, 752050 Khurda, India*

‡*Department of Applied Physics, Aalto University, FI-00076 Aalto, Finland*

¶*Varian Medical Systems Finland, FI-00270 Helsinki, Finland*

E-mail: avijitkumar@iitbbs.ac.in; peter.liljeroth@aalto.fi

Oxygen-Intercalated Graphene on Ir(111)

Figure S1 contains dI/dV spectroscopy recorded on oxygen-intercalated graphene grown on Ir(111) (G/O/Ir(111)). The dI/dV spectrum in Fig. S1a shows a phonon gap of ±80 mV which indicates phonon-mediated inelastic tunnelling process that is intrinsic to graphene layer. Fig. S1b shows field emission resonance peaks where the position of the first peak points to the fact that the work function of intercalated graphene is higher than the neutral graphene by ∼0.7 eV.

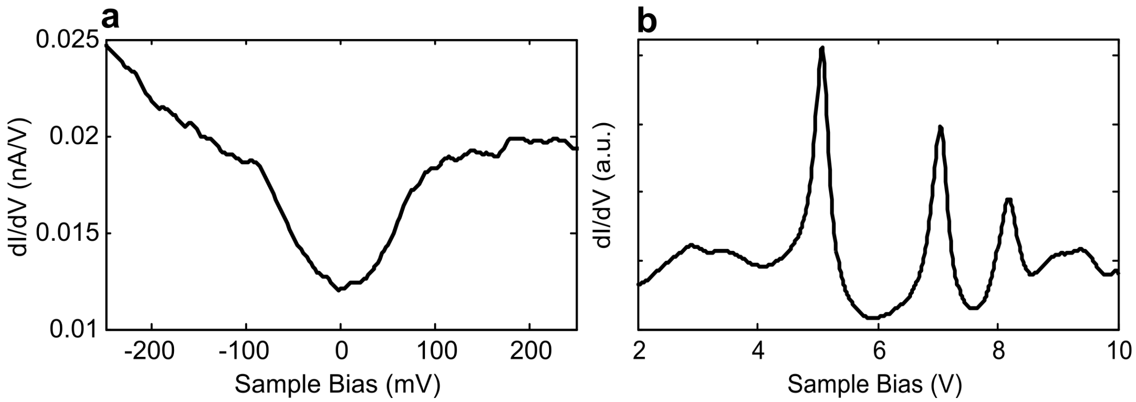

Figure S1: (a) Short range dI/dV spectrum recorded on an oxygen-intercalated graphene on Ir(111) (G/O/Ir(111)) surface shows a phonon gap of ±80 mV, which indicates that intercalated graphene is significantly decoupled.[S1] The setpoint is 10 pA at 0.5 V. (b) Field emission resonance (FER) spectrum recorded on the surface has the first peak at ∼5.0 V. This points to a work function difference of ∼0.7 eV compared to neutral graphene. This enhancement is attributed to p-type doping of graphene due to oxygen intercalation. FER spectrum was recorded at tunneling current of 0.5 nA.

Disordered Islands of CTC

Figure S2 shows an (a) overview and (b) zoomed-in STM topography images of disordered islands of CTC grown on G/O/Ir(111) surface. Disordered islands are result of sequential deposition of ∼0.25 monolayer of F_4TCNQ and TTF molecules on a G/O/Ir(111) surface at low substrate temperature (≈100 K). Annealing of such samples at room temperature for 15-45 minutes leads to the formation of highly ordered CTC islands.

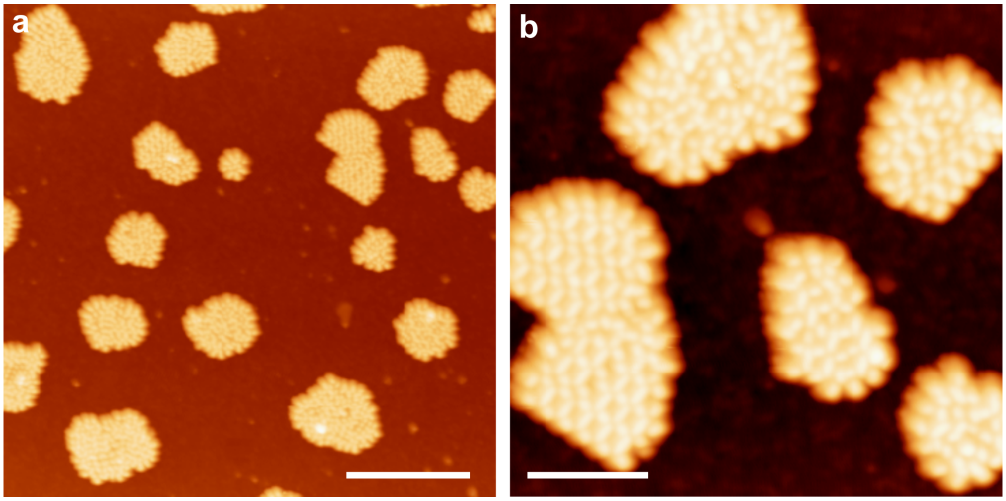

Figure S2: (a) An STM image of disordered islands of CTC on a G/O/Ir(111) surface. Imaging parameters: 0.8 pA and 1 V. The scale bar is 30 nm. (b) A zoomed-in STM image of a small area from panel (a) shows the arrangement of molecules in partially ordered and disordered islands. The bright features in the island correspond to either one or two TTF molecules. Imaging parameters: 0.8 pA and 1 V. The scale bar is 10 nm.

Checkerboard Phase of CTC

Figure S3 shows STM topography image of checkerboard phase of the CTC with stoichiometry $(F_4TCNQ)_x(TTF)_{(1-x)}$. Between two checkerboard domains, we observe regular striped domains with rows of TTF and F_4TCNQ molecules as indicated by the rectangular box.

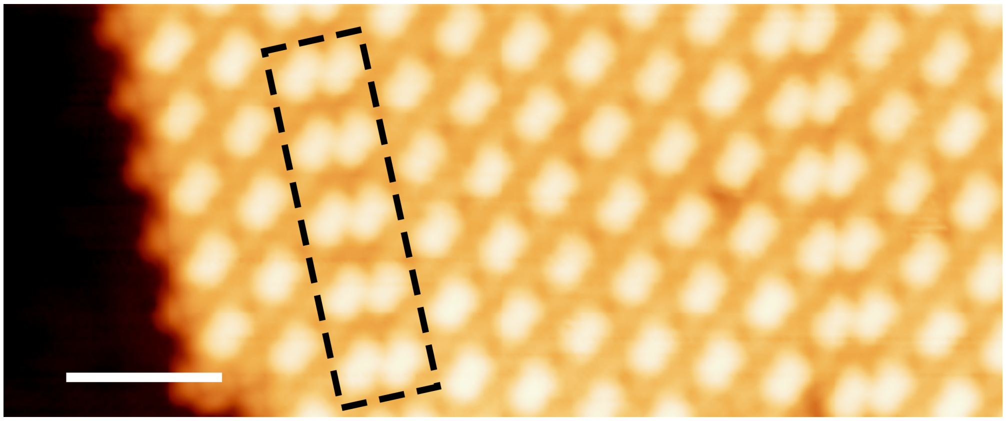

Figure S3: An STM topography image of the checkerboard phase of the CTC on a G/Ir(111) surface. The box indicates the boundary of two domains of the checkerboard phase. The boundary resembles the most common phase with alternate rows of TTF and F_4TCNQ molecules. Imaging parameters: 0.9 pA and 1.4 V. The scale bar is 3 nm.

Assembly of F_4TCNQ Molecules on G/O/Ir(111)

Figure S4a shows that single-component F_4TCNQ molecules on G/O/Ir(111) surface assemble into chains. F_4TCNQ molecules of the chain remain neutral. Figure S4b shows DFT simulated LUMO of F_4TCNQ molcule.

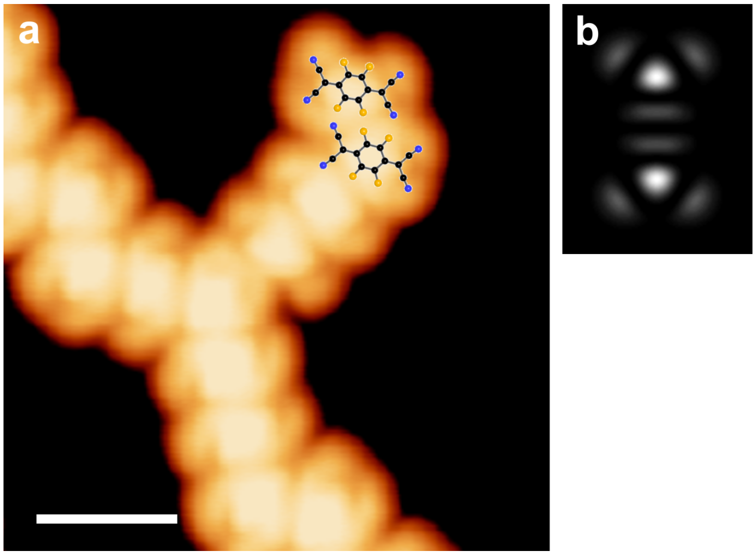

Figure S4: (a) Zig-zag arrangement of F_4TCNQ in F_4TCNQ molecular chains on a G/O/Ir(111) surface. Molecular structure of F_4TCNQ has been overlaid for clarity. Imaging parameters: 2 pA and 1.7 V. Scale bar is 2 nm. (b) A DFT simulated lowest unoccupied molecular orbital (LUMO) of F_4TCNQ.

Assembly of TTF Molecules on G/O/Ir(111)

Figure S5a shows that the single-component TTF molecules on G/O/Ir(111) assemble into a close-packed island. Similar to single-component F_4TCNQ chains on the surface, TTF molecules in the island are also neutral. Figure S5b shows DFT simulated HOMO of TTF molecule.

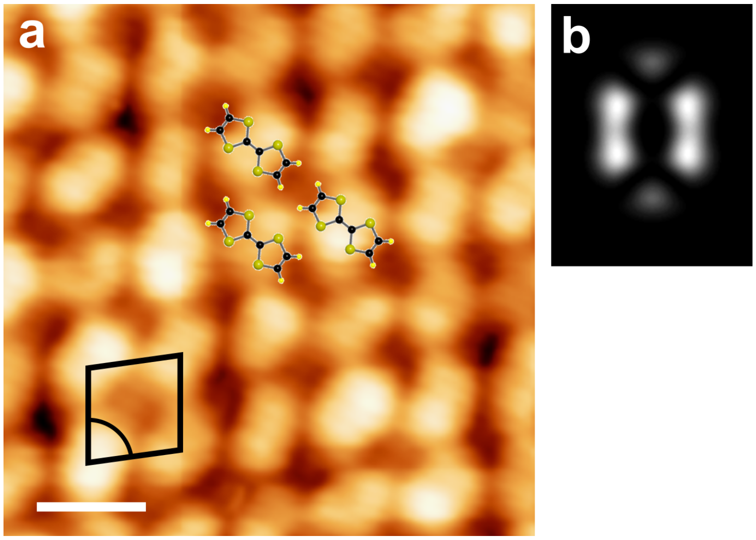

Figure S5: (a) An STM image of a small TTF island shows an oblique unit cell of the TTF assembly with unit cell of 8.0 ± 0.2 Å and angle 83°. The unit cell and molecular structure of TTF have been overlaid for clarity. Imaging parameters: 0.7 pA and 0.6 V. Scale bar is 1 nm. (b) A DFT simulated highest occupied molecular orbital (HOMO) of TTF.

Electronic Band Structure of CTC With or Without Graphene

Figure S6a shows band structure of CTC calculated using DFT with and without graphene for spin-up configuration. The band structure of the spin-down configuration (not shown) looks similar to that of the spin-up configuration. Its apparent from the band structure that electronic coupling is asymmetric in various lattice directions and the maximum width of the dispersive band is ∼150 meV. This indicates that the lateral overlap of the molecular orbitals in the CTC is relatively weak. The right panel shows total DOS of the CTC. Figure S6b is the zoomed-in plot of the same band structure.

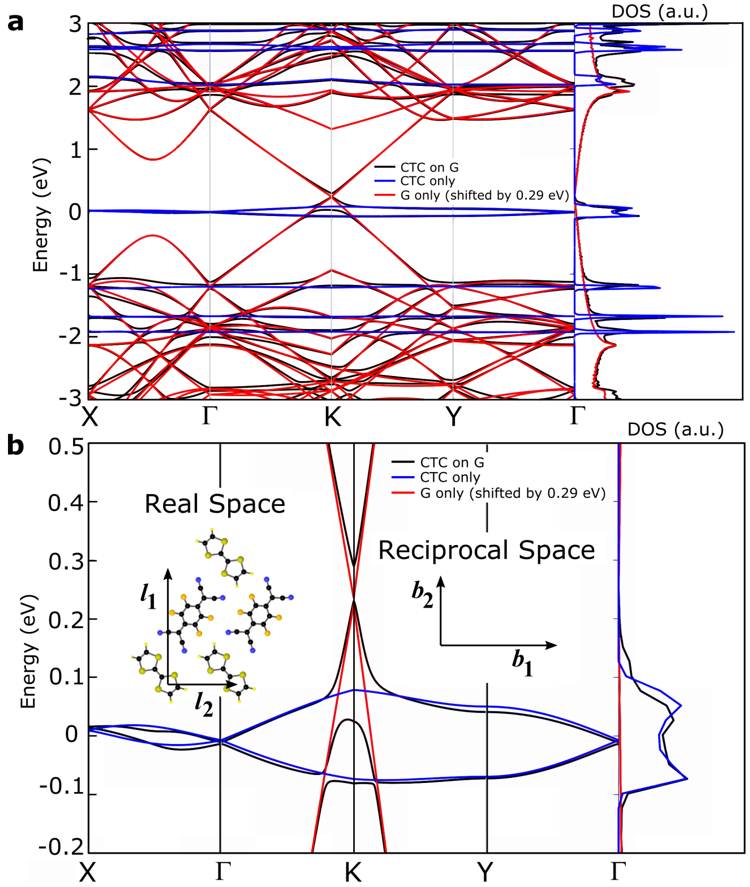

Figure S6: (a) Electronic band structure (left panel) and DOS (right panel) of CTC for its spin-up configuration. The band structure in the spin-down configuration looks very similar to that of the spin-up configuration. (b) A zoomed-in plot of the band structure from panel (a). The left and right insets show the real and the reciprocal space unit cell, respectively. The rectangular unit cell has been chosen for easier understanding of band structure in reciprocal space. Here, X = $b_1/2$ and Y = $b_2/2$.

Temperature-Dependent Spectra – Experiment and Simulated

Figure S7a contains temperature-dependent dI/dV spectra recorded on F_4TCNQ site in the CTC. The spectra has two types of features: an asymmetric dip at the Fermi energy and steps at energies ±31 meV, ±35 meV, ±52 meV. Further, the asymmetric dip can be deconvoluted into a symmetric dip and a step at the Fermi-energy. As the temperature increase the amplitude of the dip reduces and vanishes at 20 K. However, the step remains visible at temperature 20 K. Panel b shows simulated temperature dependence of dI/dV spectra assuming the the central dip has its origin due to IETS. We observe that the central dip doesn't vanish even at the temperature 20 K ruling out IETS origin of the dip.

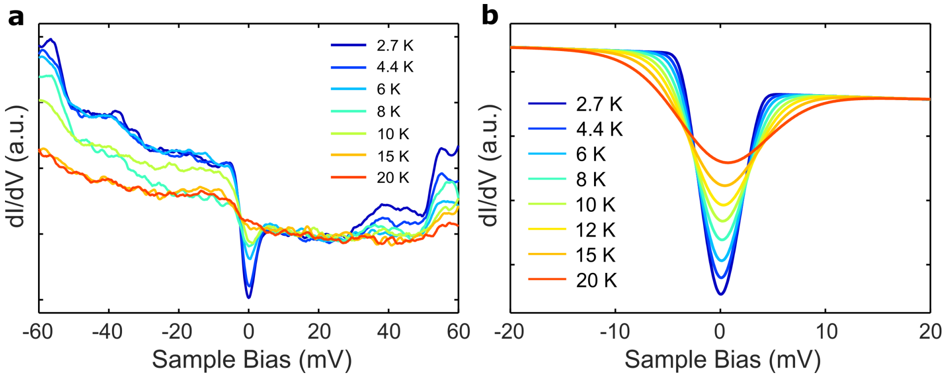

Figure S7: (a) Temperature-dependent dI/dV spectra recorded on a F_4TCNQ site in the CTC. Similar to the TTF site, the dip vanishes with increasing temperature with relatively weak asymmetry still present at 20 K. (b) Simulated temperature dependence of the dI/dV spectra assuming that the central dip would correspond to inelastic steps, where we have used the parameters extracted from the fitting of the IETS spectrum at 2.7 K. Further, each spectrum has been subjected to thermal and modulation broadening using V_{amp} = 2 mV for all spectra.

Molecular Vibrations of CTC

Figure S8 shows total DOS of the calculated modes of the CTC as function of energy and the illustration of two vibrational modes corresponding to 30 meV and 53 meV.

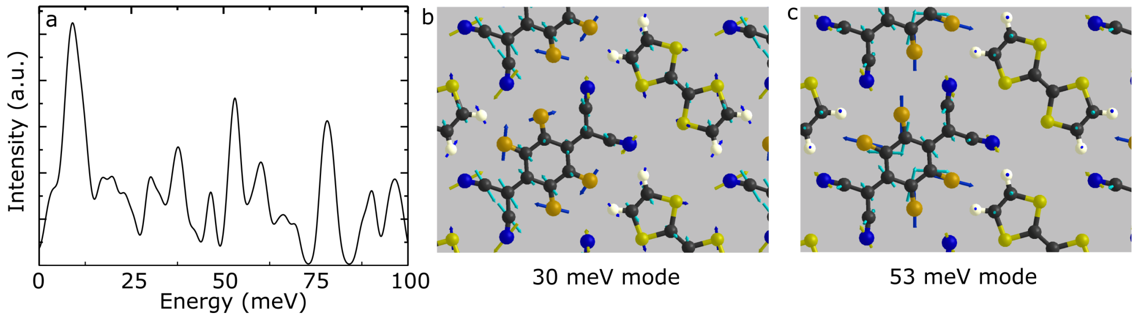

Figure S8: (a) Total DOS of the calculated vibrational modes of CTC as function of energy. (b) Vibrations in the CTC corresponding to the vibrational energy mode of 30 meV. (c) Vibrations in the CTC corresponding to the vibrational energy mode of 53 meV.

Assembly of CTC on G/Ir(111)

Figure S9 shows CTC assembly on G/Ir(111) (without oxygen intercalation) and the short-range dI/dV spectrum recorded on TTF molecule of the CTC. Here, we also observe a dip at the Fermi-energy of a comparable width as that on CTC grown on G/O/Ir(111) surface.

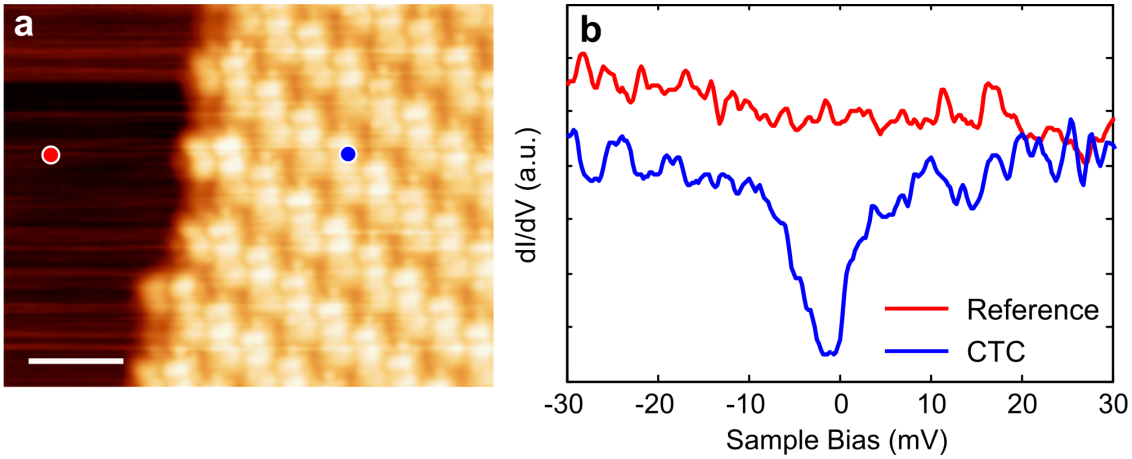

Figure S9: (a) An STM image showing a CTC assembly on graphene on a Ir(111) (G/Ir(111)) surface. Similar to that on the G/O/Ir(111) surface, the CTC has alternate rows of TTF and F_4TCNQ molecules. Imaging parameters: 10 pA and 0.1 V. Scale bar is 2 nm. (b) Short range dI/dV spectrum recorded on a TTF molecule in the CTC island shows a dip at zero sample bias. The width of the zero-bias dip is comparable to that of a CTC on G/O/Ir(111) surface. A spectrum recorded on a G/Ir(111) surface is presented as a reference.

References

(S1) Zhang, Y.; Brar, V. W.; Wang, F.; Girit, C.; Yayon, Y.; Panlasigui, M.; Zettl, A.; Crommie, M. F. Giant Phonon-Induced Conductance in Scanning Tunnelling Spectroscopy of Gate-Tunable Graphene. *Nat. Phys.* **2008**, *4*, 627.
